# Effect of Left Atrial Appendage Closure on Cardiac Function

**DOI:** 10.3390/jcdd13090425

**Published:** 2026-09-01

**Authors:** Qi Wang, Xin Li, Qi Zou, Fei Yu, Hao Hu, Xiaowei Zhang, Yujin Wang

**Affiliations:** 1 Lanzhou University Second Hospital, Lanzhou 730000, China; 2 School of Nursing, Lanzhou University, Lanzhou 730000, China; 3 Department of Cardiology, Lanzhou University Second Hospital, Lanzhou 730000, China

**Keywords:** left atrial appendage closure, atrial fibrillation, cardiac function parameters, interventional cardiology, cardiac rehabilitation

## Abstract

Atrial fibrillation (AF) is the most common clinically significant cardiac arrhythmia with a marked age-related increase in prevalence. Current guidelines recommend left atrial appendage closure (LAAC) as an alternative to long-term oral anticoagulation in selected patients at high-risk of both thromboembolic and bleeding events. Although the use of LAAC has increased substantially in recent years, its effects on cardiac function and post-procedural functional recovery remain incompletely understood. Therefore, this study aimed to evaluate changes in periprocedural cardiac function and functional parameters in patients with AF who underwent LAAC while receiving guideline-directed medical therapy. A retrospective cohort study was performed in 250 patients who underwent percutaneous LAAC at Lanzhou University Second Hospital between May 2019 and July 2025. A total of 162 patients were included in the final analysis. Echocardiographic parameters, NYHA functional class, and Barthel Index scores were compared before and after the procedure. Significant improvements were observed in several echocardiographic parameters. Post-procedural changes in NYHA functional class distribution and Barthel Index scores indicated improved functional status, with no evidence of deterioration in cardiac structure or function. These findings support the safety of LAAC with respect to cardiac functional recovery and demonstrate that the procedure does not adversely affect cardiac performance in patients with AF receiving guideline-directed medical therapy. Nevertheless, further large-scale, multi-center prospective studies are warranted to clarify the mechanisms underlying the observed improvements in cardiac function following LAAC.

## 1. Introduction

Atrial fibrillation (AF) is the most common clinically significant cardiac arrhythmia [1]. According to data from the Global Burden of Disease Study, AF has a global prevalence of approximately 2.5–3.5%, affecting an estimated 33.5 million people worldwide [2]. The pathogenesis of AF is multifactorial and involves complex hemodynamic and structural alterations. From a hemodynamic perspective, AF-induced cardiac rhythm disturbances impair ventricular filling and reduce cardiac output, ultimately leading to systemic hypoperfusion and compromised tissue oxygenation. In addition, AF alters blood flow velocity in the left atrial appendage (LAA), thereby further impairing its contractile function [3]. These changes promote blood stasis within the left atrium and facilitate thrombus formation in the LAA, thereby increasing the risk of thromboembolic complications, including stroke [4].

AF is an established independent risk factor for stroke, with LAA representing the predominant site of thrombus formation. Approximately 90% of emboli in patients with AF originate from the LAA [5,6]. Current treatment strategies focus mainly on rate control and prevention of thromboembolic events, with long-term oral anticoagulation (OAC) being the cornerstone of stroke prevention. However, this treatment is associated with an increased risk of bleeding complications, including intracranial hemorrhage and mucocutaneous bleeding [7], thus potentially contraindicated in certain patient populations. Alternative approaches have been developed to reduce the risk of ischemic stroke, including surgical LAA exclusion and percutaneous left atrial appendage closure (LAAC). LAAC is designed to prevent embolic events originating from LAA thrombi, thereby reducing the need for long-term anticoagulant therapy. Randomized controlled trials demonstrated that LAAC is non-inferior to warfarin and direct oral anticoagulants for the prevention of stroke and systemic embolism, while being associated with a lower risk of major bleeding [8]. Current ACC/AHA/ACCP/HRS guidelines recommend that LAAC should be considered as an alternative to long-term OAC in patients with non-valvular AF who are at high risk of stroke and have contraindications to prolonged anticoagulation (Class IIa recommendation, Level B evidence) [9]. The clinical use of LAAC in recent years has expanded substantially, accompanied by growing evidence supporting its role in stroke prevention [10]. Prospective studies showed that patients considered unsuitable for OAC who undergo LAAC experience lower-than-expected rates of ischemic stroke. Furthermore, registry data demonstrated high procedural success rates, low rates of major procedure-related complications, and a low incidence of ischemic stroke during follow-up [9,10,11].

AF and heart failure frequently coexist, with each condition capable of inducing or exacerbating the other through multiple pathophysiological mechanisms. Analysis of data from the Framingham Heart Study [12] demonstrated that the incidence of heart failure in AF patients is approximately threefold higher than that observed in individuals without AF. Effective management of AF is associated with improved long-term outcomes in patients with heart failure. However, the impact of LAAC on cardiac function remains controversial. Some investigators consider LAAC a purely mechanical intervention that neither terminates AF nor improves atrial electrical activity. It has been suggested that persistent AF following LAAC may contribute to a reduction in cardiac output, an increase in left atrial size and pressure, and progression of heart failure. Previous studies have suggested that left atrial appendage closure may transiently affect neuroendocrine status through altered production of neurohormones from the left atrium, raising a theoretical concern that reduced ANP levels might adversely affect sodium and water balance [13]. However, the net clinical impact remains controversial. In contrast, other studies reported significant improvements in cardiac functional parameters following LAAC. Given these conflicting findings, the present retrospective cohort study was performed to evaluate changes in periprocedural cardiac functional parameters and rehabilitation-related outcomes in patients with AF who underwent LAAC while receiving guideline-directed medical therapy. Furthermore, post-procedural cardiac functional recovery was assessed through the comparison of NYHA functional class and Barthel Index scores obtained during follow-up with corresponding preoperative baseline values.

Occluders currently used in clinical practice can be broadly classified as plug-type devices (e.g., Watchman and Watchman FLX) or disc-type devices (e.g., LAmbre and Laxible). Both designs are intended to conform to LAA anatomy and minimize residual peri-device shunting. Although the number and variety of available LAAC devices continue to increase, evidence comparing their respective effects on cardiac functional recovery remains limited. Therefore, this study also aimed to evaluate the potential differential effects of disc-type and plug-type occluders on post-procedural cardiac functional parameters.

## 2. Materials and Methods

### 2.1. Study Population

A retrospective cohort study was performed in 250 patients who underwent percutaneous LAAC at Lanzhou University Second Hospital between May 2019 and July 2025. A total of 162 eligible patients were included in the final analysis following the application of the predefined inclusion and exclusion criteria. Of these, 39 patients (24.07%) received disc-type occluders and 123 (75.93%) received plug-type occluders.

### 2.2. Inclusion Criteria

(1) Confirmed diagnosis of non-valvular AF with guideline-recommended indications for LAAC and successful device implantation.
(2) Availability of complete preoperative and post-procedural clinical and imaging data.
(3) Patients undergoing an isolated LAA occlusion procedure.

### 2.3. Exclusion Criteria

(1) Severe hepatic insufficiency (Child–Pugh class C) or renal insufficiency (eGFR < 30 mL/min/1.73 m^2^).
(2) Presence of LAA or atrial thrombus on pre-procedural transesophageal echocardiography.
(3) Severe hyperthyroidism (TSH < 0.1 μIU/mL with symptomatic thyrotoxicosis).
(4) Inability to complete follow-up because of cognitive impairment or geographical barriers.
(5) Patients undergoing radiofrequency ablation.

**Figure 1:**
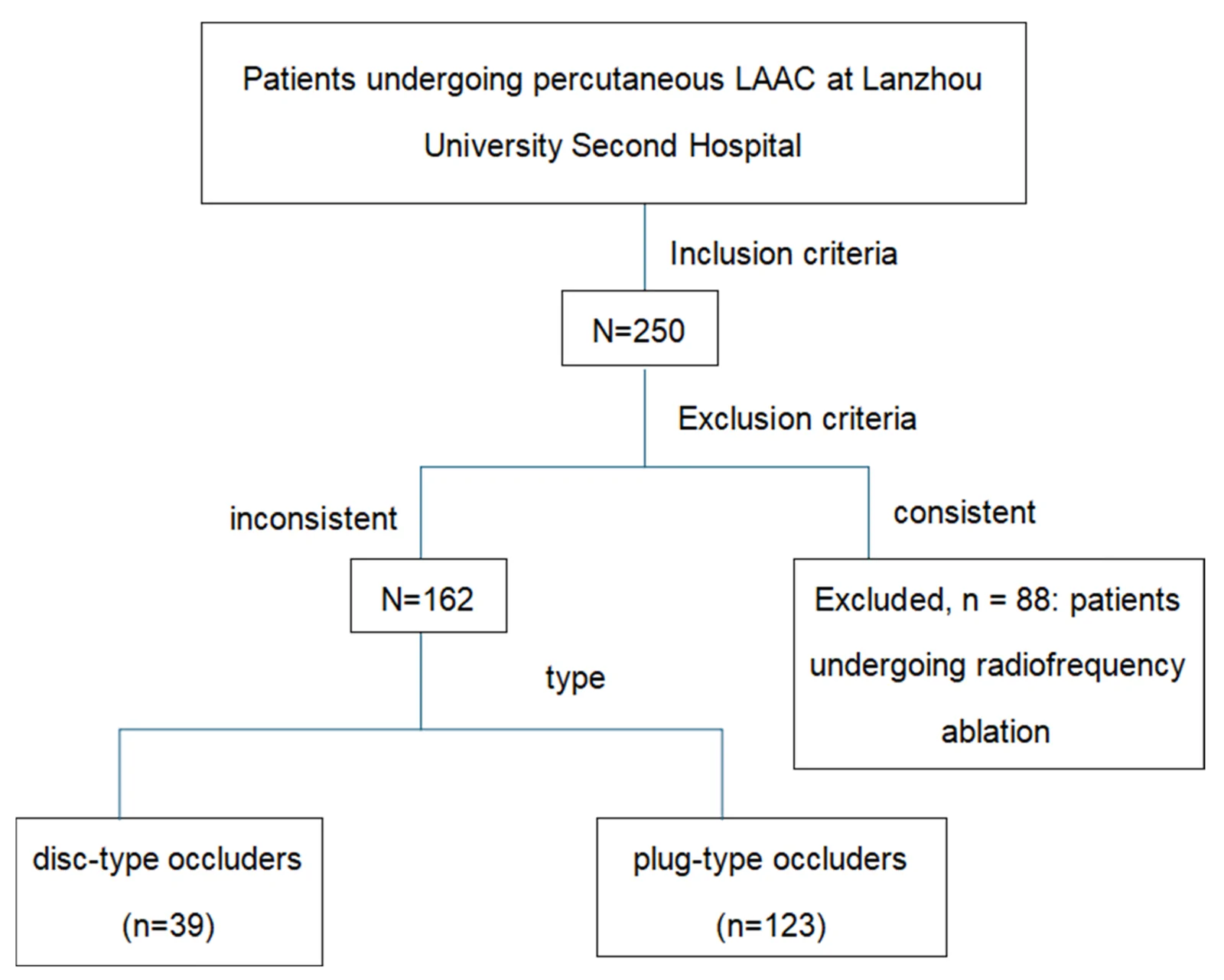
Flowchart illustrating patient selection, exclusion criteria, and allocation according to occluder type.

The patient selection process, including the inclusion and exclusion criteria, is summarized in Figure 1.

### 2.4. Data Collection and Outcome Assessment

#### 2.4.1. Collection of Baseline Characteristics

Patients’ demographic and baseline clinical characteristics were collected through a systematic review of medical records. Data included sex, age, height, weight, body mass index (BMI, calculated as weight [kg]/height [m]^2^); comorbidities (hypertension, diabetes mellitus, coronary artery disease, chronic heart failure, history of bleeding disorders, and history of cerebrovascular accident/transient ischemic attack (TIA)); liver and renal functional status; and the type of implanted occluder. In addition, systolic and diastolic blood pressure data were recorded at baseline and at the 6-month follow-up. All enrolled patients received guideline-directed medical therapy according to contemporary recommendations before the procedure. In addition, CHA_2_DS_2_-VASc and HAS-BLED scores were recorded and analyzed.

The CHA_2_DS_2_-VASc score includes the following 9 indicators: congestive heart failure (1 point), hypertension (1 point), age ≥ 75 years (2 points), diabetes mellitus (1 point), previous stroke/TIA or thromboembolism (2 points), age 65–74 years (1 point), female sex (1 point), and vascular disease (1 point). A score of ≥2 in male patients or ≥3 in female patients is generally considered indicative of an elevated thromboembolic risk and supports the use of anticoagulant therapy.

The HAS-BLED score includes the following 9 indicators: hypertension, renal dysfunction, hepatic dysfunction, previous stroke, bleeding tendency or hemorrhagic diathesis, unstable international normalized ratio (INR), age > 65 years, concomitant use of antiplatelet agents or non-steroidal anti-inflammatory drugs (NSAIDs), and alcohol abuse. Each indicator is assigned 1 point, yielding a maximum total score of 9. A score of ≥3 indicates a high bleeding risk, while a score of ≤2 indicates a relatively low bleeding risk.

#### 2.4.2. Collection of Echocardiographic Parameters

This retrospective study retrieved echocardiographic data, including both preoperative baseline and post-procedural follow-up information, from the hospital’s Picture Archiving and Communication System. For patients who underwent LAAC, a follow-up transthoracic echocardiography (TTE) was performed at 6 months post-procedure. Eligible examinations were performed using a standardized ultrasound platform between May 2019 and July 2025, with adequate image quality and complete data availability. All examinations were performed by experienced cardiac sonographers according to the department’s standard protocol. Structural parameters included left atrial diameter (LAD), left ventricular end-diastolic diameter (LVEDD), and left ventricular end-systolic diameter (LVESD). Systolic function was assessed using left ventricular ejection fraction (LVEF, Simpson’s biplane method), stroke volume (SV), cardiac output (CO), and fractional shortening (FS). Diastolic and right heart function were evaluated using the diastolic function index E/e’ ratio and pulmonary artery systolic pressure (PASP).

#### 2.4.3. Collection of NYHA Classification and Barthel Index

Preoperative NYHA functional class and Barthel Index scores were obtained from patients’ medical records. Post-procedural assessment was performed during follow-up at least 6 months after the procedure.

The NYHA classification is a physician-assigned measure of functional limitation that ranges from Class I (no symptoms during normal physical activity) to Class IV (symptoms at rest).

The Barthel Index consists of 10 items assessing activities of daily living and may be scored using different systems. In the version used in this study, individual items are scored as 0, 5, 10, or 15, resulting in a total score ranging from 0 to 100, with higher scores indicating greater functional independence. The Barthel Index was employed as an exploratory tool to quantify changes in activities of daily living before and after LAAC. Although our study population was not severely functionally dependent, the Barthel Index remains a validated and widely used instrument that can capture subtle changes in functional status, including post-procedural recovery of basic self-care abilities. It was intended to complement the NYHA classification, which primarily reflects cardiovascular functional capacity, by providing a patient-centered perspective on actual daily performance.

### 2.5. Statistical Analysis

Statistical analysis was performed using SPSS Statistics version 27.0 (IBM Corp., Armonk, NY, USA). Continuous variables are presented as mean ± standard deviation (SD), while categorical variables are presented as frequencies and percentages. Baseline characteristics were compared between the plug-type and disc-type occluder groups using independent-samples *t*-tests for continuous variables and χ^2^ tests for categorical variables. Changes in echocardiographic parameters before and after LAAC were evaluated using paired-samples *t*-tests. Missing continuous data were handled using median imputation to preserve sample size and minimize the influence of extreme values. For comparison of NYHA functional class before and after LAAC, the Wilcoxon signed-rank test was employed since these data were derived from the same patients and represented paired ordinal categorical variables. A two-sided value of *p* < 0.05 was considered statistically significant.

## 3. Results

### 3.1. Baseline Characteristics

A total of 162 patients with non-valvular AF were included. Overall, the study cohort was characterized by advanced age, high thromboembolic (CHA_2_DS_2_-VASc score ≥ 2) and bleeding risk, and high prevalence of cardiovascular comorbidities, consistent with the typical clinical population undergoing LAA occlusion. Baseline characteristics are summarized in Table 1.

### 3.2. Comparison of Baseline Characteristics with Different Occluders

Among the 162 patients included in this study, 123 received plug-type occluders, and 39 received disc-type occluders. No significant differences were observed between the two groups with respect to demographic characteristics or baseline clinical parameters. The mean age was 66.8 ± 9.2 years in the plug-type group and 65.4 ± 7.4 years in the disc-type group (*p* = 0.386). Mean BMI was 24.7 ± 3.5 kg/m^2^ and 25.5 ± 3.8 kg/m^2^, respectively (*p* = 0.240). The mean CHA_2_DS_2_-VASc score was 3.4 ± 1.8 in the plug-type group and 3.8 ± 1.5 in the disc-type group (*p* = 0.281), while the corresponding HAS-BLED scores were 2.2 ± 1.4 and 2.6 ± 1.2, respectively (*p* = 0.210).

The prevalence of major comorbidities was comparable between the two groups. Hypertension was present in 61.8% and 71.8% of patients in the plug-type and disc-type groups, respectively (*p* = 0.256). No statistically significant between-group difference was observed in the prevalence of diabetes mellitus (22.0% vs. 28.2%, *p* = 0.422), coronary heart disease (30.9% vs. 35.9%, *p* = 0.560), previous stroke/TIA (29.3% vs. 28.2%, *p* = 0.899), heart failure (36.6% vs. 23.1%, *p* = 0.119), liver dysfunction (7.3% vs. 12.8%, *p* = 0.287), or history of bleeding (7.3% vs. 7.7%, *p* = 0.938).

Overall, the plug-type and disc-type occluder groups were well balanced with respect to baseline demographic characteristics, thromboembolic and bleeding risk scores, and major comorbidities (all *p* > 0.05).

The baseline characteristics of the two groups are summarized in Table 1.

### 3.3. Comparison of Cardiac Functional Parameters

Significant changes were observed in all assessed echocardiographic parameters following LAAC compared with preoperative values (all *p* < 0.05). Specifically, LAD decreased from 42.4 ± 6.3 mm to 41.5 ± 6.3 mm (*p* = 0.043), LVEF increased from 60.2 ± 8.2% to 62.4 ± 8.2% (*p* = 0.001), and the E/e’ ratio decreased from 14.4 ± 8.7 to 11.6 ± 3.6 (*p* = 0.034). In addition, LVESD decreased from 32.0 ± 6.0 mm to 31.0 ± 5.7 mm (*p* = 0.005); LVEDD decreased from 47.5 ± 5.8 mm to 46.8 ± 5.6 mm (*p* = 0.049), and PASP decreased from 35.8 ± 10.1 mmHg to 34.1 ± 10.3 mmHg (*p* = 0.026). Measures of systolic function also improved, with FS increasing from 32.1 ± 5.8% to 33.6 ± 5.6% (*p* = 0.002), SV increasing from 62.3 ± 14.3 mL to 65.0 ± 17.3 mL (*p* = 0.040), and CO increasing from 5.2 ± 1.3 L/min to 5.5 ± 1.5 L/min (*p* = 0.027). Systolic blood pressure showed a mild increasing trend (116.4 ± 10.7 mmHg vs. 118.2 ± 11.9 mmHg), while diastolic blood pressure showed a slight decrease (70.4 ± 6.6 mmHg vs. 70.1 ± 6.9 mmHg); however, neither change reached statistical significance (*p* = 0.060 and *p* = 0.596, respectively).

The comparison of NYHA functional class distributions before and after LAAC was statistically significant (*p* = 0.017). Most patients were classified as NYHA class II before surgery (81 patients, 50.0%) or class III (50 patients, 30.9%), whereas class I accounted for 24 (14.8%) and class IV for 7 (4.3%) patients. Class II remained the predominant category (87 patients, 53.7%) following surgery, while the proportion of class III patients decreased to 18.5% (30 patients) and the proportion of class I patients increased to 22.8% (37 patients). The proportion of class IV patients remained largely unchanged (8 patients, 4.9%). Overall, the post-procedural distribution shifted towards lower NYHA functional classes. Barthel Index scores significantly increased from 84.2 ± 10.7 before surgery to 87.90 ± 8.9 after LAAC (*p* < 0.001), indicating improved performance in activities of daily living. Detailed cardiac function metrics are presented in Table 2.

### 3.4. Comparison of Cardiac Functional Parameters in the Heart Failure Subgroup

In the heart failure subgroup (*n* = 54), significant post-procedural improvements were observed in LVEF (57.2 ± 11.0% vs. 60.5 ± 10.8%, *p* = 0.020), FS (30.1 ± 7.2% vs. 32.8 ± 6.7%, *p* = 0.007), SV (59.2 ± 14.0 mL vs. 66.4 ± 20.6 mL, *p* = 0.005), and LVESD (33.6 ± 8.6 mm vs. 32.1 ± 7.9 mm, *p* = 0.034). LAD showed a borderline decrease (44.8 ± 5.8 mm vs. 43.7 ± 6.0 mm, *p* = 0.051). E/e′ (17.5 ± 14.9 vs. 12.5 ± 4.8, *p* = 0.226), LVEDD (48.6 ± 7.7 mm vs. 47.8 ± 7.7 mm, *p* = 0.160), PASP (38.3 ± 11.5 mmHg vs. 37.5 ± 12.2 mmHg, *p* = 0.600), and CO (4.9 ± 1.3 L/min vs. 5.2 ± 1.3 L/min, *p* = 0.233) all showed improving trends but did not reach statistical significance. NYHA class distribution improved, with Class I increasing from 9.3% to 24.1% and Class III + IV decreasing from 40.8% to 29.6%, approaching statistical significance (*p* = 0.073). The Barthel Index also improved significantly (80.5 ± 12.6 vs. 85.7 ± 12.0, *p* = 0.008). Detailed results are presented in Table 3.

### 3.5. Changes in Cardiac Function Parameters Following Different Occluder Device Implantations

Subgroup analysis according to occluder type showed comparable baseline characteristics between plug-type and disc-type groups. Improvements in systolic function parameters were observed in both groups following LAAC. LVEF increased from 60.0 ± 8.6 to 62.0 ± 8.4 in the plug-type group, while FS increased from 32.0 ± 6.0 to 33.5 ± 5.8 (both *p* < 0.05). Similarly, LVEF increased from 60.8 ± 6.7 to 63.5 ± 7.5 in the disc-type group, and FS increased from 32.2 ± 5.2 to 34.2 ± 5.3 (both *p* < 0.05). Although numerically greater improvements in LVEF and FS were observed in the disc-type group, the differences between the two groups did not reach statistical significance (*p* > 0.05). Overall, neither occluder type was associated with impaired left ventricular systolic function. Detailed results are presented in Table 4.

## 4. Discussion

AF is independently associated with increased mortality and major cardiovascular events, including ischemic stroke and TIA. AF-related abnormality in atrial electrical activity may impair both systolic and diastolic cardiac function, resulting in irregular ventricular contraction and an increased risk of heart failure [7]. Current management strategies emphasize stroke prevention and integrated patient care. In this context, LAAC has emerged as an alternative to long-term OAC for selected patients with AF, providing effective stroke prevention while reducing the risk of bleeding associated with chronic anticoagulant therapy [14]. It utilizes an occluder device to seal off the LAA, thereby preventing thrombi formed within the LAA from entering the systemic circulation via blood flow. This plays a critical role in stroke prevention.

Although current ACC/AHA/ACCP/HRS guidelines support the use of LAAC as an alternative to long-term anticoagulation [9], its effect on cardiac remodeling and functional recovery remains controversial. Phan et al. [13] reported reductions in LVEF and FS following LAAC compared with non-LAAC controls. The authors suggested that these changes may be related to the loss of the LAA’s contribution to left ventricular volume regulation and left atrial pressure buffering. In contrast, Luani et al. [15] found no significant changes in LVEF at either 6 weeks or 6 months after successful LAAC. However, several studies reported favorable cardiac remodeling following the procedure. Mitrega et al. [16] observed improvements in left ventricular function, including increases in LVEF and SV at 3 months after LAAC. Similarly, Coisne et al. [17] demonstrated improvements in left atrial expansion index and LAEF. The findings of the present study are generally consistent with these reports, supporting the concept that LAAC does not adversely affect cardiac function and may be associated with favorable functional and structural changes.

This study demonstrated significant changes in cardiac structure and function following LAAC. Both LVESD and LVEDD decreased significantly compared with preoperative values, indicating that left ventricular dimension did not increase after the procedure. Furthermore, LAD decreased significantly, suggesting attenuation of atrial remodeling. These findings are consistent with previous reports indicating that reverse remodeling may occur following LAAC without deterioration of cardiac function [18]. LAA exclusion may contribute to favorable hemodynamic changes by reducing blood stasis within the atrium and modifying left atrial loading conditions. Consistent with this hypothesis, PASP decreased significantly after the procedure, reflecting a reduction in right ventricular afterload. During atrial fibrillation, the LAA loses effective contractile function but retains passive distensibility and pressure-buffering capacity. LAA closure achieves stroke prevention by excluding this structure; however, the net hemodynamic effect represents a balance between thrombus elimination and the loss of physiological functions, rather than a simple reduction in left atrial volume load. LAAC excludes this stagnant volume from circulation, thereby directly reducing the volume load on the left atrium. Furthermore, the E/e′ ratio, an established indicator of left ventricular filling pressure and diastolic function, was decreased significantly after LAAC, suggesting reduced left ventricular filling pressure without evidence of worsening diastolic function. Notably, blood pressure remained relatively stable throughout the follow-up period, with only mild, non-significant changes observed in either systolic or diastolic values (both *p* > 0.05). This suggests that the observed improvements in cardiac functional parameters were unlikely to be attributed to blood pressure fluctuations, further supporting the association between LAAC and favorable functional recovery. Beyond its passive mechanical properties, the LAA also contributes to left ventricular filling through passive elastic recoil during early diastole, particularly in patients with preserved left ventricular function. Following LAA exclusion, this contribution is eliminated, which may theoretically affect stroke volume and cardiac output. However, in the present cohort, we observed no reduction in these parameters, suggesting that compensatory mechanisms—such as improved filling time and reduced heart rate—may have offset the loss of LAA passive filling. Similarly, the loss of LAA compliance may initially increase left atrial pressure sensitivity to volume changes; the lack of observed hemodynamic deterioration in our patients may reflect successful long-term compensatory adaptation involving left atrial and pulmonary venous remodeling.

We further performed a subgroup analysis of patients with concomitant heart failure. The results demonstrated that post-procedural LVEF, FS, SV, and LVESD did not deteriorate in this subgroup, while NYHA classification showed an improving trend, and the Barthel Index improved significantly, indicating that LAAC was not associated with further functional impairment even in patients with compromised baseline cardiac function. This finding carries important clinical implications, as patients with coexisting heart failure and atrial fibrillation generally have a poorer prognosis and more limited treatment options. The observation that LAAC does not impair cardiac function provides additional safety evidence for its clinical application in this high-risk population. Nevertheless, given the relatively small sample size of the heart failure subgroup (*n* = 54), some parameter changes did not reach statistical significance. These findings should therefore be considered exploratory and warrant confirmation in larger-scale studies.

Previous studies [19] reported that chronic AF affects ventricular mechanics and contributes to progressive cardiac remodeling. The present findings suggested that LAAC did not negatively affect these processes. Instead, the observed improvements in structural and hemodynamic parameters supported the possibility of favorable cardiac adaptation following the procedure. Overall, the present results indicated that LAAC was not associated with deterioration in cardiac structure, systolic function, or hemodynamic status in this cohort. In addition to the observed echocardiographic improvements, post-procedural functional assessment suggested that LAAC was not associated with deterioration in patients’ overall functional status. Significant improvements were observed in NYHA functional class distribution and Barthel Index scores following the procedure. The shift toward lower NYHA functional classes indicated a reduction in symptom burden and improved functional capacity. Similarly, the increase in Barthel Index scores suggested enhanced performance in activities of daily living and greater functional independence after LAAC. These findings complement the observed improvements in objective cardiac parameters and support the conclusion that LAAC did not adversely affect post-procedural rehabilitation. Instead, the procedure was associated with preservation or improvement of both cardiac function and functional status in this cohort. This virtuous cycle of “symptom relief → increased confidence → more activity → improved capacity” represents the core objective of post-procedural rehabilitation for patients. Such a positive feedback loop helps strengthen patients’ confidence in and adherence to treatment, which may, in turn, lead to long-term clinical prognostic benefits. In summary, the absence of deterioration in subjective symptom burden and the maintenance of objective daily living ability indicate that patients’ overall functional status and quality of life were not adversely affected following the procedure.

The observed post-procedural values of LVEF, FS, and SV suggest that left ventricular systolic function was not adversely affected following LAAC, which may be associated with alterations in atrial natriuretic peptide (ANP) and brain natriuretic peptide (BNP) levels. Previous studies [13] showed that alterations in atrial natriuretic peptide (ANP) and brain natriuretic peptide (BNP) secretions may contribute to post-procedural cardiac adaptation. The LAA functions not only as a structural component of the left atrium but also as a neuroendocrine organ. Approximately 30% of total ANP production is secreted by the LAA [5]. Naya et al. [20] demonstrated that percutaneous LAAC significantly affects natriuretic peptide release, with a marked reduction in circulating ANP and BNP observed shortly after device implantation. Similarly, Phan et al. [13] reported decreased ANP secretion following LAA exclusion, accompanied by alterations in salt and water homeostasis. These studies demonstrate that LAAC significantly suppresses its neurohumoral secretory function, resulting in reduced plasma levels of ANP and BNP. This attenuation of neuroendocrine regulation exerts a dual effect: it modulates water-sodium balance by decreasing diuretic hormone release, and it lowers peripheral vascular resistance. Together, these mechanisms reduce cardiac preload and afterload, leading to significant hemodynamic improvement and enhanced cardiac function in patients. Although these observations suggest a potential role for neurohumoral mechanisms in the cardiac changes observed after LAAC, the present study did not systematically analyze circulating ANP and BNP levels. Given the established role of these peptides in ventricular remodeling and fluid homeostasis, future investigations should prioritize serial ANP/BNP measurements to quantify the relationship between neurohormonal shifts and observed functional improvements and elucidate potential mechanisms underlying LAAC-mediated cardiac recovery.

Transesophageal echocardiography (TEE) is used for the assessment of LAA anatomy, including ostial size, morphology, and depth, thereby assisting surgeons in the selection of an appropriate size of occluder device [21]. The Watchman device is the first LAA occluder approved for clinical use internationally. The PROTECT-AF and PREVAIL trials [22] demonstrated that WATCHMAN implantation is associated with reduced risk of hemorrhagic stroke, disabling or fatal stroke, and major bleeding events compared with oral anticoagulation. The LAmbre occluder exhibits a significantly lower risk of LAA injury compared with the Watchman device, while no statistically significant differences were observed between the two types of LAA occluders in terms of procedural success, deployment success, or device repositioning requirements. These findings suggest that both device types can achieve effective LAA closure with acceptable procedural safety profiles. In the present study, both plug-type and disc-type occluders were associated with significant improvements in cardiac functional parameters after LAAC. Although numerically greater increases in LVEF and FS were observed in the disc-type group, the between-group differences did not reach statistical significance. Therefore, the current findings did not support the conclusion that one occluder type was superior to the other with respect to post-procedural cardiac functional recovery. Anatomical mismatch between the typically elliptical LAA ostium and certain occluder designs may influence sealing performance and contribute to peri-device leakage (PDL) [23]. Comparative analyses [24] reported lower PDL rates with disc-type devices than with plug-type devices, potentially reflecting differences in device structure and sealing mechanisms. These differential sealing characteristics may subsequently influence the trajectory of post-procedural cardiac functional recovery in patients. In addition, procedural sequencing and device-specific implantation strategies may influence post-procedural outcomes and procedural performance [25]. However, whether these factors translate into clinically meaningful differences in cardiac functional recovery remains uncertain and warrants further investigation.

## 5. Conclusions

This retrospective cohort study demonstrated that LAAC was not associated with deterioration in cardiac structure, diastolic function, functional status, or activities of daily living among patients receiving guideline-directed medical therapy. Significant improvements were observed in several echocardiographic parameters, together with favorable changes in NYHA functional class. These findings support the clinical safety of LAAC and suggest that the procedure does not adversely affect post-procedural cardiac rehabilitation. Further prospective multicenter studies with systematic biomarker assessments are required to clarify the mechanisms underlying the observed functional changes and validate these findings in larger patient populations.

## Figures and Tables

**Table 1:** Baseline characteristics.

	Total (*n* = 162)	Plug-Type (*n* = 123)	Disc-Type (*n* = 39)	*p*
Age (years)	66.5 ± 8.8	66.8 ± 9.2	65.4 ± 7.4	0.386
Height (cm)	167.7 ± 7.7	167.5 ± 7.8	168.4 ± 7.5	0.492
Weight (kg)	70.2 ± 11.9	69.4 ± 11.3	72.7 ± 13.4	0.134
BMI (kg/m^2^)	24.9 ± 3.6	24.7 ± 3.5	25.5 ± 3.8	0.240
CHA_2_DS_2_-VASc (scores)	3.5 ± 1.7	3.4 ± 1.8	3.8 ± 1.5	0.281
HAS-BLED (scores)	2.3 ± 1.4	2.2 ± 1.4	2.6 ± 1.2	0.210
Female, *n* (%)	61 (37.7%)	47 (38.2%)	14 (35.9%)	0.795
Hypertension, *n* (%)	104 (64.2%)	76 (61.8%)	28 (71.8%)	0.256
Diabetes mellitus, *n* (%)	38 (23.5%)	27 (22.0%)	11 (28.2%)	0.422
History of bleeding, *n* (%)	12 (7.4%)	9 (7.3%)	3 (7.7%)	0.938
Liver dysfunction, *n* (%)	14 (8.6%)	9 (7.3%)	5 (12.8%)	0.287
Renal dysfunction, *n* (%)	10 (6.2%)	8 (6.5%)	2 (5.1%)	0.756
Coronary heart disease, *n* (%)	52 (32.1%)	38 (30.9%)	14 (35.9%)	0.560
Heart failure, *n* (%)	54 (33.3%)	45 (36.6%)	9 (23.1%)	0.119
History of stroke/TIA, *n* (%)	47 (29.0%)	36 (29.3%)	11 (28.2%)	0.899

**Table 2:** Comparative analysis of cardiac functional parameters pre- and post-LAAC in AF patients (*n* = 162).

		Preoperative	Post-Procedural	*p*
LAD		42.4 ± 6.3	41.5 ± 6.3	0.043 *
EF		60.2 ± 8.2	62.4 ± 8.2	<0.001 *
E/e		14.4 ± 8.7	11.6 ± 3.6	0.034 *
LVESD		32.0 ± 6.0	31.0 ± 5.7	0.005 *
LVEDD		47.5 ± 5.8	46.8 ± 5.6	0.049 *
PASP		35.8 ± 10.1	34.1 ± 10.4	0.026 *
FS		32.1 ± 5.8	33.6 ± 5.7	0.002 *
SV		62.3 ± 14.3	65.0 ± 17.3	0.040 *
CO		5.2 ± 1.3	5.5 ± 1.5	0.027 *
Systolic blood pressure (mmHg)		116.4 ± 10.7	118.2 ± 11.9	0.060
Diastolic blood pressure (mmHg)		70.4 ± 6.6	70.1 ± 6.9	0.596
NYHA Classification Criteria	Class I	24 (14.82%)	37 (22.84%)	0.017 *
	Class II	81 (50.00%)	87 (53.70%)	
	Class III	50 (30.86%)	30 (18.52%)	
	Class IV	7 (4.32%)	8 (4.94%)	
Barthel index		84.2 ± 10.7	87.9 ± 8.9	<0.001 *

* *p* < 0.05.

**Table 3:** Comparative analysis of cardiac functional parameters pre- and post-LAAC in AF patients (*n* = 54).

		Preoperative	Post-Procedural	*p*
LAD		44.8 ± 5.8	43.7 ± 6.0	0.051
EF		57.2 ± 11.0	60.5 ± 10.8	0.020 *
E/e		17.5 ± 14.9	12.5 ± 4.8	0.226
LVESD		33.6 ± 8.6	32.1 ± 7.9	0.034 *
LVEDD		48.6 ± 7.7	47.8 ± 7.7	0.160
PASP		38.3 ± 11.5	37.5 ± 12.2	0.600
FS		30.1 ± 7.2	32.8 ± 6.7	0.007 *
SV		59.2 ± 14.0	66.4 ± 20.6	0.005 *
CO		4.9 ± 1.3	5.2 ± 1.3	0.233
NYHA Classification Criteria	Class I	5 (9.3%)	37 (24.1%)	0.073
	Class II	27 (50%)	87 (46.3%)	
	Class III	15 (27.8%)	30 (18.5%)	
	Class IV	7 (13.0%)	8 (11.1%)	
Barthel index		80.5 ± 12.6	85.7 ± 12.0	0.008 *

* *p* < 0.05.

**Table 4:** Preoperative and post-procedural cardiac function parameters and changes in the plug-type and disc-type occluder groups.

	Plug Occluder			Disc Occluder			Between-Group Comparison
	Preoperative	Post-Procedural	*p*	Preoperative	Post-Procedural	*p*	*p* (Δ Between Groups)
LAD	42.3 ± 6.3	41.3 ± 6.4	0.050 *	42.5 ± 6.4	41.9 ± 6.1	0.527	0.720
EF	60.0 ± 8.6	62.0 ± 8.4	0.009 *	60.8 ± 6.7	63.5 ± 7.5	0.030 *	0.681
E/e	15.1 ± 9.7	11.7 ± 3.7	0.046 *	12.2 ± 3.3	11.4 ± 3.1	0.277	0.835
LVESD	32.1 ± 6.3	31.3 ± 6.0	0.065	31.7 ± 5.0	30.0 ± 4.2	0.014 *	0.204
LVEDD	47.5 ± 6.0	47.2 ± 5.8	0.349	47.5 ± 5.3	45.8 ± 5.1	0.045 *	0.131
PASP	36.0 ± 10.4	34.5 ± 10.9	0.104	35.4 ± 9.0	32.9 ± 8.4	0.084	0.594
FS	32.0 ± 6.0	33.5 ± 5.8	0.015 *	32.2 ± 5.3	34.2 ± 5.3	0.025 *	0.656
SV	62.2 ± 14.4	65.3 ± 16.7	0.029 *	62.6 ± 14.2	64.0 ± 19.4	0.666	0.628
CO	5.1 ± 1.3	5.5 ± 1.6	0.056	5.2 ± 1.1	5.6 ± 1.3	0.273	0.822

* *p* < 0.05.

## Data Availability

Data availability is restricted due to ongoing research; the data presented in this study can be obtained from the corresponding author upon reasonable request.

## Abbreviations

The following abbreviations are used in this manuscript:

AF	atrial fibrillation
LAAC	left atrial appendage closure
LVEF	left ventricular ejection fraction
FS	fractional shortening
SV	stroke volume
OAC	oral anticoagulation
HF	heart failure
CHF	congestive heart failure
HR	hazard ratios
LAA	left atrial appendage
BMI	body mass index
TIA	transient ischemic attack
INR	international normalized ratio
TTE	transthoracic echocardiography
LAD	left atrial diameter
PASP	pulmonary artery systolic pressure
SD	standard deviation
LVESD	left ventricular end-systolic diameter
LVEDD	left ventricular end-diastolic diameter
CA	catheter ablation
ANP	atrial natriuretic peptide
BNP	brain natriuretic peptide
PDL	peri-device leakage
